# Subpopulation treatment effect pattern plot analysis: a prognostic model for distant recurrence-free survival to estimate delayed adjuvant chemotherapy initiation effect in triple-negative breast cancer

**DOI:** 10.3389/fonc.2023.1193927

**Published:** 2023-11-06

**Authors:** Zaida Morante, Yomali Ferreyra, Joseph A. Pinto, Natalia Valdivieso, Carlos Castañeda, Tatiana Vidaurre, Guillermo Valencia, Patricia Rioja, Hugo Fuentes, José M. Cotrina, Silvia Neciosup, Henry L. Gomez

**Affiliations:** ^1^ Departamento de Medicina Oncológica, Instituto Nacional de Enfermedades Neoplásicas, Lima, Peru; ^2^ Oncosalud, AUNA, Lima, Peru; ^3^ Departamento de Bioingeniería, Universidad de Ingeniería y Tecnología, Lima, Peru; ^4^ Centro de Investigación Básica y traslacional, Auna Ideas, Lima, Peru; ^5^ Departamento de Cirugía en Mamas y Tejidos Blandos, Instituto Nacional de Enfermedades Neoplásicas, Lima, Peru; ^6^ Instituto de Investigaciones en Ciencias Biomédicas (INICIB), Universidad Ricardo Palma, Lima, Peru

**Keywords:** breast cancer, subpopulation treatment effect pattern plot, triple negative breast cancer, adjuvant chemotherapy, prognostic factor analysis

## Abstract

**Introduction:**

Triple-negative breast cancer (TNBC) is a heterogeneous disease associated with a poor prognosis. Delaying in time to start adjuvant chemotherapy (TTC) has been related to an increased risk of distant recurrence-free survival (DRFS). We aimed to develop a prognostic model to estimate the effects of delayed TTC among TNBC risk subgroups.

**Materials and methods:**

We analyzed 687 TNBC patients who received adjuvant chemotherapy at the Instituto Nacional de Enfermedades Neoplasicas (Lima, Peru). Database was randomly divided to create a discovery set (n=344) and a validation set (n=343). Univariate and multivariate Cox regression models were performed to identify prognostic factors for DRFS. Risk stratification was implemented through two models developed based on proportional hazard ratios from significant clinicopathological characteristics. Subpopulation treatment effect pattern plot (STEPP) analysis was performed to determine the best prognostic cut-off points for stratifying TNBC subgroups according to risk scores and estimate Kaplan-Meier differences in 10-year DRFS comparing TTC (≤30 vs.>30 days).

**Results:**

In univariate analysis, patients aged ≥70 years (HR=4.65; 95% CI: 2.32-9.34; p=<0.001), those at stages pT3-T4 (HR=3.28; 95% CI: 1.57-6.83; p=0.002), and pN2-N3 (HR=3.00; 95% CI: 1.90-4.76; p=<0.001) were notably associated with higher risk. STEPP analysis defined three risk subgroups for each model. Model N°01 categorized patients into low (score: 0–31), intermediate (score:32–64), and high-risk (score: 65–100) cohorts; meanwhile, Model N°02: low (score: 0–26), intermediate (score: 27–55), and high (score: 56–100). Kaplan-Meier plots showed that in the discovery set, patients with TTC>30 days experienced a 17.5% decrease in 10-year DRFS rate (95%CI=6.7-28.3), and the impact was more remarkable in patients who belong to the high-risk subgroup (53.3% decrease in 10 years-DRFS rate). Similar results were found in the validation set.

**Conclusions:**

We developed two prognostic models based on age, pT, and pN to select the best one to classify TNBC. For Model N°02, delayed adjuvant chemotherapy conferred a higher risk of relapse in patients ≥70 years and who were characterized by pT3/T4 and pN2/N3. Thus, more efforts should be considered to avoid delayed TTC in TNBC patients, especially those in high-risk subgroups.

## Introduction

Triple-negative breast cancer (TNBC) is a heterogeneous and complex disease characterized by the absence of estrogen receptors (ER), progesterone receptors (PR), and HER2 amplification (1, 2). TNBC could be classified into six different subtypes regarding transcriptional profiling: two basal-like (BL1 and BL2), an immunomodulatory (IM), a mesenchymal (M), a mesenchymal stem-like (MSL), and a luminal AR (LAR) subtype (3).

TNBC represents 10%-20% of all BCs worldwide (4, 5), which amounts to nearly 200,000 cases each year (6). A high prevalence of TNBC has been reported among Hispanic, Afro-American, premenopausal, or carriers of BRCA gene mutations (7, 8). In Latin America, the incidence of TNBC has been described in several countries, such as Mexico (23.1%), Venezuela (24.6%), Brazil (27%), and Peru (21.3%) (9, 10). TNBC patients from these countries usually face socioeconomic barriers to timely access to specialized cancer treatment (1, 11, 12).

TNBC also exhibits a higher probability of early tumor relapse, metastases, and death than patients with other BC subtypes (13, 14). Despite advances in BC treatments, including targeted therapies and immunotherapies, the management of TNBC remains particularly challenging. Unlike hormone receptor-positive or HER2-positive subtypes, TNBC lacks well-defined molecular targets for therapy, limiting the treatment options mainly to chemotherapy (5).

For TNBC patients, treatment mainly consists of surgery and chemotherapy (15). Adjuvant chemotherapy is the standard of care for this BC subtype because it helps to reduce the risk of a distant recurrence and improves overall survival (OS) (16, 17). Yu et al. (2013) reported that the 5-year OS for TNBC patients receiving adjuvant chemotherapy was 85% compared to 76% in those who did not (HR=0.65; 95% CI=0.52-0.81; p<0.001) (18).

In recent years, delayed initiation of adjuvant chemotherapy (TTC) has been associated with a worse prognosis (5). Among TNBC patients, TTC > 4 weeks has been associated with an 89% increase in the risk of recurrence (95% CI= 1.09-3.27; p=0.024) and a 2.49 times increase in the risk of death (95%CI= 1.30-4.76; p=0.006) compared to those with TTC ≤ 4 weeks (18).

Considering the limited access to treatment and irregular outcomes for TNBC patients, conducting studies to select patients who promptly need adjuvant chemotherapy is essential. A Subpopulation Treatment Effect Pattern Plot (STEPP) analysis is an alternative approach to estimating interactions between treatment and a continuous covariate of interest (19). Kaplan-Meier plots provided by this method allow defining cut-off points to identify subpopulations that may respond differently to a treatment. By stratifying patients into risk groups, STEPP can provide insights into personalized treatment decisions, helping optimize the benefit-risk balance for each patient.

In this current study, we aimed to develop a simple and easy-to-use prognostic model based on clinical characteristics to evaluate the effect of TTC on relapse risk among TNBC subgroups.

## Materials and methods

### Design and study population

A retrospective study reported 2007 cases of TNBC diagnosed between January 2000 and December 2014 at the National Institute of Neoplastic Diseases (INEN) in Lima, Peru. Immunohistochemistry (IHC) and fluorescent *in situ* hybridization (FISH) techniques were utilized to identify TNBC patients.

Antibodies Estrogen Anti-Receptor (Clone 1D5, Dako), Progesterone Anti-Receptor (Clone PGR636, Dako), and Anti-HER2/neu (A0485, Dako) were used to assess IHC analysis. A tumor was defined as negative for either ER or PR if less than 10% of its cells showed any level of nuclear staining. HER2 status was determined following the American Society of Clinical Oncology and College of American Pathologists (ASCO-CAP) guidelines. FISH results corroborated the absence of HER2 protein overexpression. More details about the cohort design have already been reported in previous literature (20).

### Eligibility criteria

We only included TNBC patients (stage I to III) who underwent surgery as the first treatment and then received adjuvant chemotherapy. Male patients, inflammatory breast tumor cases, and patients lacking information about tumor size, surgery type, chemotherapy, or surgery dates were excluded. TNBC patients treated with neoadjuvant chemotherapy were not considered in this study.

### Definition of variables

Medical records were reviewed to obtain information on clinical variables such as age, diagnosis period (2000 – 2004, 2005 – 2009, 2010 – 2014), pathological tumor size (pT) and lymph nodes (pN), histological grade, vascular permeability, and treatment completion. Incomplete chemotherapy was defined as patients receiving less than four cycles of anthracyclines. DRFS was estimated in years from the initial intervention (surgery) to the date of documentation of distant recurrence or last contact with the patients. TTC was calculated by the days between surgery and the first dose of adjuvant chemotherapy. TTC variable was: ≤30 and >30 days.

### Statistical analysis

Database was randomly divided to create a discovery (n=344) and validation set (n=343). Discovery set was used to calculate the proportional hazard ratios strongly associated with DRFS. Univariate and multivariate Cox regression models were conducted to create adjusted models. Clinical characteristics included in those models, such as age, pT and pN, had to be statistically significant in univariate and multivariate Cox analyses. Subsequently, log-rank tests were performed to determine whether HRs from the variables selected were constant over time.

Linear models were developed based on the summation of proportional hazard ratios from the clinicopathological characteristics of each patient (21, 22). Risk scores from all patients were scaled from 0 to 100. STEPP method was used to define appropriate cut-off values to split patients into groups with different DRFS probabilities and estimate the effect of TTC on those risk subgroups, which were based on Kaplan-Meier estimations of 10-year DRFS (y-axis) throughout the composite risk score (x-axis). In previous research, STEPP was also used to determine cut-off values and divided patients with diffuse and/or intestinal-type gastric cancer in risk groups for peritoneal recurrence using R (23). STEPP parameters had a window size of 160 (r2) and 30 (r1) patients with a recommended number of permutations of 2500 for stable results (24). Standard errors at 95%CI were considered to assess the distant recurrence effect.

Subsequently, survival analyses were performed to determine whether the subgroups created by the STEPP analysis were statistically significant to DRFS. Additionally, composite risk values from the discovery and validation sets were compared to clinical stages using multivariate Cox analysis to evaluate which represents a major significant risk in DRFS. Data were analyzed with R software version 4.0.3 using the packages “survival” (version 3.5-5), “survminer” (version 0.4.9), and “stepp” (version 3.2.5).

### Ethical considerations

The study was approved by the Ethics Review Board of the Instituto Nacional de Enfermedades Neoplasicas (INEN 22-03) and conducted in compliance with all relevant ethical guidelines.

## Results

### General characteristics of TNBC patients

In this study, a total of 687 TNBC patients were included. Median age of the population was 48.0 (IQR: 41.0, 57.0) years. Most of them were determined to have a pT2 (62.6%), pN0 (48.8%), and G3 (81.0%). A higher proportion of patients were found to be aged 41-59 years (n=388, 56.5%) and diagnosed between 2005-2009 (n=287, 41.8%). Similar trends were reported in the discovery set (n=344) and validation set (n=343; **Table 1**).

**Table 1:** General characteristics of TNBC patients.

Clinicopathologic characteristics	Overall, N = 687^1^	Discovery set, N = 344^1^	Validation set, N = 343^1^
**Age (years)**	48.0 (41.0, 57.0)	49.0 (41.0, 57.0)	48.0 (40.5, 56.5)
Age groups (years)			
≤ 40	165 (24.0%)	79 (23.0%)	86 (25.1%)
41 – 59	388 (56.5%)	197 (57.3%)	191 (55.7%)
≥ 60	134 (19.5%)	68 (19.8%)	66 (19.2%)
Age groups (years)			
≤ 40	165 (24.0%)	79 (23.0%)	86 (25.1%)
41 – 59	388 (56.5%)	197 (57.3%)	191 (55.7%)
60 – 69	98 (14.3%)	49 (14.2%)	49 (14.3%)
≥ 70	36 (5.2%)	19 (5.5%)	17 (5.0%)
Diagnostic period			
2000 – 2004	195 (28.4%)	94 (27.3%)	101 (29.4%)
2005 – 2009	287 (41.8%)	144 (41.9%)	143 (41.7%)
2010 – 2014	205 (29.8%)	106 (30.8%)	99 (28.9%)
pT			
pT1	110 (17.1%)	50 (15.7%)	60 (18.5%)
pT2	402 (62.6%)	198 (62.3%)	204 (63.0%)
pT3/T4	130 (20.2%)	70 (22.0%)	60 (18.5%)
pTX	45	26	19
pN			
pN0	317 (48.8%)	162 (50.5%)	155 (47.3%)
pN1	205 (31.6%)	96 (29.9%)	109 (33.2%)
pN2/N3	127 (19.6%)	63 (19.6%)	64 (19.5%)
pNX	38	23	15
Histological grade			
I/II	122 (19.0%)	60 (18.8%)	62 (19.3%)
III	519 (81.0%)	260 (81.3%)	259 (80.7%)
Not reported	46	24	22
Vascular permeability			
No	284 (49.7)	142 (50.0)	142 (49.3)
Yes	288 (50.3%)	142 (50.0%)	146 (50.7%)
Not reported	115	60	55
Incomplete chemotherapy			
No	537 (80.0)	274 (81.3)	263 (78.7)
Yes	134 (20.0%)	63 (18.7%)	71 (21.3%)
Not reported	16	7	9
TTC (days)			
≤ 30	188 (27.4%)	84 (24.4%)	104 (30.3%)
> 30	499 (72.6%)	260 (75.6%)	239 (69.7%)

^1^Median (IQR); n (%).

### Univariate and multivariate Cox analysis

Univariate and multivariate Cox analysis for clinicopathological characteristics related to DRFS showed that only age, pT, and pN were significantly associated with recurrence (**Table 2**). Both age stratifications were significant for the outcome. For Univariate analysis, clinicopathologic characteristics that contributed to a higher risk for recurrence were age ≥ 70 years (vs. ≤40/41-59/60-69; HR=4.65; 95%CI:2.32-9.34; p=<0.001), stage pT3-T4 (vs. pT1/T2; HR=3.28; 95%CI:1.57-6.83; p=0.002), followed by pN2-N3 (vs. pN0/N1; HR=3.00, 95%CI:1.90-4.76; p=<0.001) and age ≥ 60 years (vs. ≤40/41-59; HR=2.38; 95%CI:1.36-4.18; p=0.003; **Table 2**). Evaluation of the proportional hazards (PH) assumption determined that HRs of the three characteristics (age, pT, and pN) were constant over time (**Figure S1**).

**Table 2:** Univariate and multivariate analysis of TNBC patients and characteristics clinicopathologic related to DRFS in the discovery set.

Characteristic	Univariate analysis				Multivariate analysis (adjusted model 1)				Multivariate analysis (adjusted model 2)			
	N	HR^1^	95% CI^1^	p-value	PE^2^	HR^1^	95% CI^1^	p-value	PE^2^	HR^1^	95% CI^1^	p-value
Age groups (years)												
≤ 40	79	—	—			—	—					
41 – 59	197	1.30	0.78, 2.19	0.314	0.34	1.40	0.81, 2.44	0.233				
≥ 60	68	2.38	1.36, 4.18	**0.003**	0.98	2.67	1.46, 4.88	**0.001**				
Age groups (years)												
≤ 40	79	—	—							—	—	
41 – 59	197	1.31	0.78, 2.19	0.3					0.34	1.40	0.80, 2.43	0.2
60 – 69	49	1.78	0.95, 3.34	0.071					0.73	2.07	1.05, 4.08	**0.036**
≥ 70	19	4.65	2.32, 9.34	**<0.001**					1.44	4.20	2.04, 8.66	**<0.001**
Diagnostic period												
2000 – 2004	94	—	—									
2005 – 2009	144	1.43	0.91, 2.24	0.12								
2010 – 2014	106	0.98	0.57, 1.70	>0.9								
pT												
pT1	50	—	—			—	—			—	—	
pT2	198	1.93	0.96, 3.88	0.065	0.58	1.79	0.88, 3.61	0.11	0.51	1.67	0.82, 3.38	0.2
pT3/T4	70	3.28	1.57, 6.83	**0.002**	1.07	2.92	1.39, 6.17	**0.005**	1.00	2.71	1.28, 5.75	**0.009**
pN												
pN0	162	—	—			—	—			—	—	
pN1	96	1.74	1.09, 2.78	**0.019**	0.61	1.85	1.15, 2.96	**0.011**	0.60	1.83	1.14, 2.94	**0.012**
pN2/N3	63	3.00	1.90, 4.76	**<0.001**	1.02	2.78	1.74, 4.45	**<0.001**	1.00	2.71	1.69, 4.34	**<0.001**
Histological grade												
I/II	60	—	—									
III	260	0.85	0.54, 1.36	0.5								
Vascular permeability												
No	142	—	—									
Yes	142	1.42	0.95, 2.13	0.090								
Incomplete chemotherapy												
No	274	—	—									
Yes	63	1.53	0.99, 2.37	0.055								

^1^HR, Hazard Ratio; CI, Confidence Interval.

^1^PE, Parameter; Estimated, ln (HR).

Bold values mean that p-values are statistically significant (p<0.05).

The symbol "—" means that it is the reference variable for Cox analysis.

### Prognostic models

The median follow-up period for the discovery set was 9.6 years. Regression models were described according to proportional hazard ratios from the Multivariate analyses:


$$Composite\;risk\;score\;{\left(Model\;N{}^{\circ}1\right)}_{i}=\;(\begin{array}{c} 0.00\\ 0.34\\ 0.98 \end{array}\begin{array}{c} ,\;\\ ,\\ , \end{array}\begin{array}{c} {\text{ age}}_{\text{i}}\;\leq \;40\\ \;41\leq {\text{age}}_{\text{i}}\leq 59\\ {\text{ age}}_{\text{i}}\;\geq 60 \end{array})+\;(\begin{array}{c} 0.00\\ 0.58\\ 1.07 \end{array}\begin{array}{c} ,\;\\ ,\\ , \end{array}\begin{array}{c} {\text{ pT}}_{\text{i}}=\text{pT}1\\ {\text{ pT}}_{\text{i}}=\text{pT}2\\ {\text{ pT}}_{\text{i}}=\text{pT}3-\text{T}4 \end{array})+\;(\begin{array}{c} 0.00\\ 0.61\\ 1.02 \end{array}\begin{array}{c} ,\;\\ ,\\ , \end{array}\begin{array}{c} {\text{ pN}}_{\text{i}}=\text{pN}0\\ {\text{ pN}}_{\text{i}}=\text{pN}1\\ {\text{ pN}}_{\text{i}}=\text{pN}2-\text{N}3 \end{array})$$

$$Composite\;risk\;score\;{\left(Model\;N{}^{\circ}2\right)}_{i}=\;(\begin{array}{ccc} 0.00 & , & {\text{ age}}_{\text{i}}\;\leq \;40\\ 0.34 & , & \;41\leq {\text{age}}_{\text{i}}\leq 59\\ \begin{array}{c} 0.73\\ 1.44 \end{array} & \begin{array}{c} ,\\ , \end{array} & \begin{array}{c} \;60\leq {\text{age}}_{\text{i}}\leq 69\\ {\text{ age}}_{\text{i}}\;\geq 70 \end{array} \end{array})+\;(\begin{array}{c} 0.00\\ 0.51\\ 1.00 \end{array}\begin{array}{c} ,\;\\ ,\\ , \end{array}\begin{array}{c} {\text{ pT}}_{\text{i}}=\text{pT}1\\ {\text{ pT}}_{\text{i}}=\text{pT}2\\ {\text{ pT}}_{\text{i}}=\text{pT}3-\text{T}4 \end{array})+\;(\begin{array}{c} 0.00\\ 0.60\\ 1.00 \end{array}\begin{array}{c} ,\;\\ ,\\ , \end{array}\begin{array}{c} {\text{ pN}}_{\text{i}}=\text{pN}0\\ {\text{ pN}}_{\text{i}}=\text{pN}1\\ {\text{ pN}}_{\text{i}}=\text{pN}2-\text{N}3 \end{array})$$

Equations indicated that the composite risk scores could vary according to the profile of each patient (*i*). Scores of all patients were scaled from 0 to 100, where 100 implied the patient obtained the maximum value of the equation. According to the STEPP methodology, patients were stratified into three subgroups based on their composite risk values.

**Figure 1:**
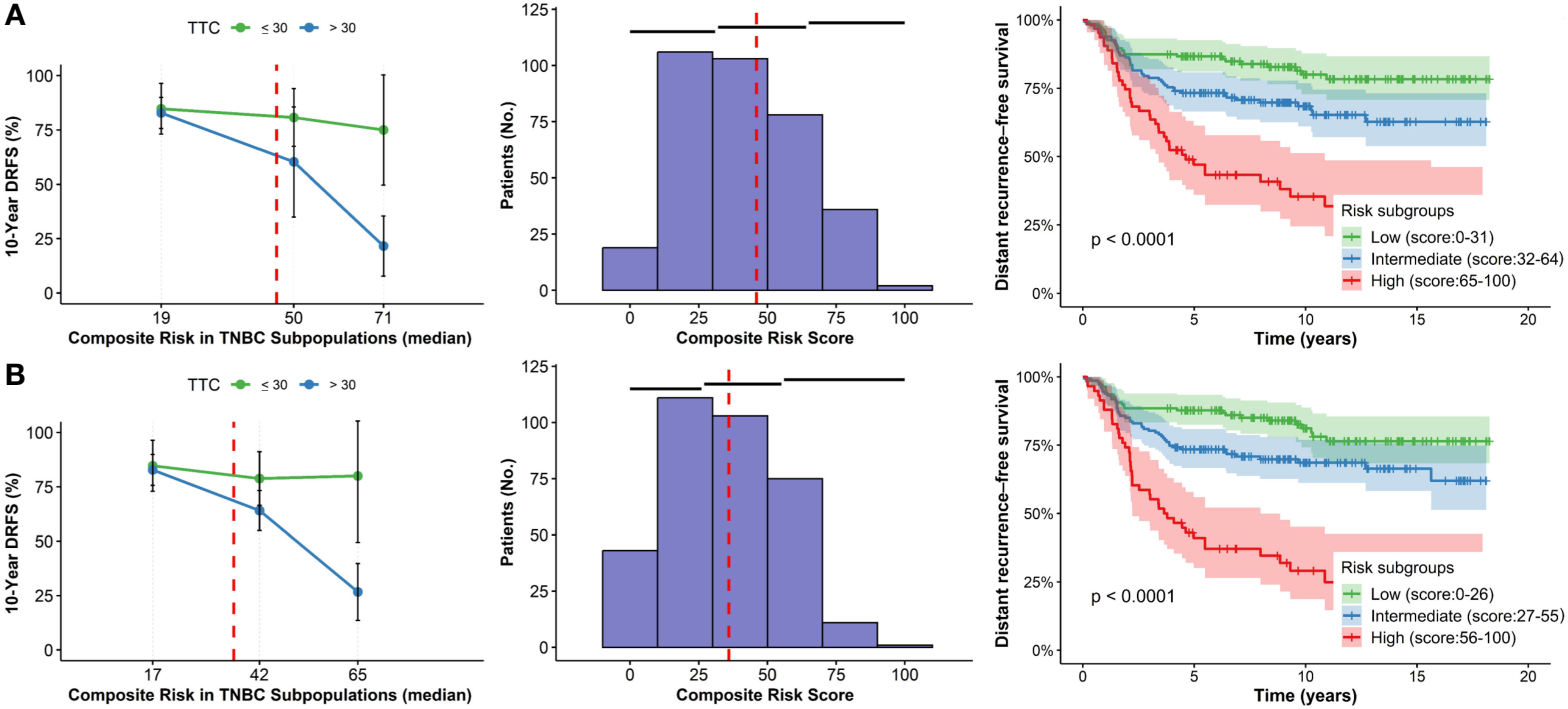
STEPP plots of 10-year DRFS, histograms and Kaplan-Meier curves according to composite risk scores in subgroups according to TTC for the discovery set. **(A)** Model N°01. **(B)** Model N°2.

For Model N°01, the median composite risk values for the discovery set were 46 (IQR: 30, 55). Low (score: 0 – 31), intermediate (score: 32 – 64), and high risk (score: 65 – 100) (**Figure 1A**; **Table 3**). For Model N°02, the median composite risk values were 36 (IQR: 25, 53) and also could classify patients with a low (score: 0 – 26), intermediate (score: 27 – 55), and high risk (score: 56 – 100) (**Figure 1B**; **Table 3**). The overall decrease for 10-year DRFS among patients who started adjuvant treatment >30 days vs. those who started ≤ 30 days was 17.5% ± 10.8% for both models (**Table 3**).

**Table 3:** 10-year DRFS (%). For each analysis, results show three subpopulations at either extreme of the STEPP (lowest and highest composite risk values) as well as the intermediate STEPP subpopulations for both discovery and validation sets.

Risk subgroups (Model N°01)	Discovery set			Validation set		
	≤ 30 days	> 30 days	Δ ± SE (95% CI)	≤ 30 days	> 30 days	Δ ± SE (95% CI)
Low (score: 0 – 31)	84.7 ± 11.7	82.8 ± 7.1	- 1.9 ± 13.7	86.9 ± 9.3	81.6 ± 7.8	- 5.3 ± 12.1
Intermediate (score: 32 – 64)	78.8 ± 13.3	64.1 ± 8.8	- 14.6 ± 16.0	78.0 ± 11.7	63.9 ± 9.4	- 14.1 ± 15.0
High (score: 65 – 100)	80.0 ± 25.3	26.7 ± 13.8	**- 53.3 ± 28.8**	84.6 ± 20.0	40.8 ± 16.7	**- 43.8 ± 26.0**
** Overall effect**	81.1 ± 8.8	63.8 ± 6.3	- 17.5 ± 10.8	82.6 ± 7.5	66.6 ± 6.5	- 16.0 ± 9.9
Risk subgroups (Model N°02)	Discovery set			Validation set		
	≤ 30 days	> 30 days	Δ ± SE (95% CI)	≤ 30 days	> 30 days	Δ ± SE (95% CI)
Low (score: 0 – 26)	84.7 ± 11.7	82.8 ± 7.1	- 1.9 ± 13.7	86.9 ± 9.3	81.6 ± 7.8	- 5.3 ± 12.1
Intermediate (score: 27 – 55)	80.7 ± 12.3	60.3 ± 9.2	- 20.4 ± 15.3	78.0 ± 11.7	63.9 ± 9.6	- 14.1 ± 15.2
High (score: 56 – 100)	75.0 ± 30.6	21.6 ± 13.1	**- 53.4 ± 49.9**	58.8 ± 23.9	36.1 ± 13.9	**- 22.7 ± 27.6**
**Overall effect**	81.3 ± 8.7	63.8 ± 6.3	- 17.5 ± 10.8	82.6 ± 7.5	66.6 ± 6.5	- 16.0 ± 9.9

Bold values are the maximum values in the column.

However, the effects may vary depending on each subpopulation created by the STEPP methodology. For Model N°01, it could be observed that patients who belong to the higher risk subgroup and started chemotherapy ≤30 days experienced an increment in 53.3% ± 28.8% of 10-year DRFS vs. those who initiated adjuvant >30 days (**Table 3**). Alternatively, in the low-risk subgroup, the DRFS variation was marginal (1.9% ± 13.7%), implying that those with lower risk characteristics in the composite value have similar effects even if they started their adjuvant treatment ≤30 days or >30 days. In Model N°02, the high-risk subgroup initiating chemotherapy within the same temporal frame experienced a 10-year DRFS of 53.4% ± 49.9%, also in contrast to those who initiated treatment later (**Table 3**). Low-risk subgroup also displayed minimal changes in DRFS, regardless of TTC. In both models, high-risk individuals delaying treatment beyond 30 days could be considered critical regarding the prognostic risk of recurrence.

In Model N°01, Cox Univariate analysis revealed significant risk stratification among patients into three subgroups: Low vs. Intermediate (HR=1.90; 95% CI= 1.17-3.08; p=0.010) and Low vs. High (HR=4.58; 95% CI=2.77-7.56; p=<0.001; **Figure 1A**; **Table 4**). Similarly, in Model N°02, significant risk differentiation was observed: Low vs. Intermediate (HR=1.82; 95% CI=1.13-2.95; p=0.015) and Low vs. High (HR=5.29; 95% CI=3.22-8.69; p=<0.001; **Figure 1B**; **Table 4**).

**Table 4:** Univariate Cox analysis of TNBC patients’ distant recurrence in discovery and validation set regarding risk subgroups.

Risk subgroups (Model N°01)	Discovery set				Validation set			
	N	HR^1^	95% CI^1^	p-value	N	HR^1^	95% CI^1^	p-value
Low (score: 0 – 31)	135	—	—		137	—	—	
Intermediate (score: 32 – 64)	146	1.90	1.17, 3.08	**0.010**	148	2.03	1.25, 3.31	**0.005**
High (score: 65 – 100)	63	4.58	2.77, 7.56	**<0.001**	58	3.50	2.03, 6.05	**<0.001**
Risk subgroups (Model N°02)	Discovery set				Validation set			
	N	HR^1^	95% CI^1^	p-value	N	HR^1^	95% CI^1^	p-value
Low (score: 0 – 26)	139	—	—		134	—	—	
Intermediate (score: 27 – 55)	147	1.82	1.13, 2.95	**0.015**	160	2.48	1.49, 4.15	**<0.001**
High (score: 56 – 100)	58	5.29	3.22, 8.69	**<0.001**	49	4.97	2.79, 8.89	**<0.001**

Bold values mean that p-values are statistically significant (p<0.05).

The symbol "—" means that it is the reference variable for Cox analysis.

### Validation analysis

**Figure 2:**
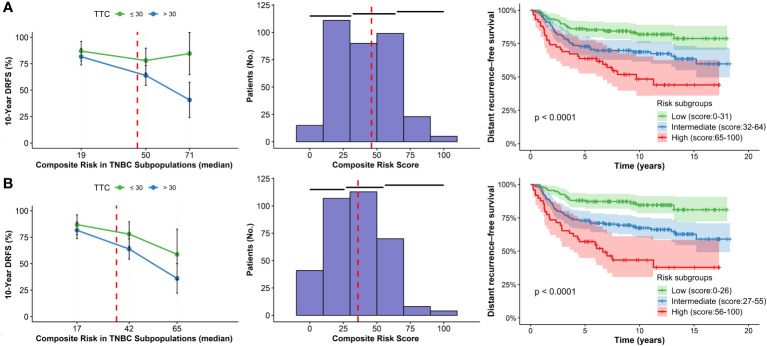
STEPP plots of 10-year DRFS, histograms and Kaplan-Meier curves according to composite risk scores in subgroups according to TTC for the validation set. **(A)** Model N°01. **(B)** Model N°2.

The median follow-up period for the validation set was 10.1 years, and regarding its composite risk values, for Model N°01, the median score was 46 (IQR: 30 - 55). Regarding Model N°02, the median score was 36 (IQR: 25 - 49). Cut-off points for the risk groups from both models were the same as in the discovery set (**Figure 2**; **Table 3**).

In Model N°01, high-risk patients initiating chemotherapy after 30 days exhibited a 43.8% ± 26.0% absolute decrease in 10-year DRFS rate compared to those who started before 30 days (**Table 3**). For the low-risk population with delayed treatment, the rate dropped by 5.3% ± 12.1%. Conversely, in Model N°02, the high-risk patients experienced a 22.7% ± 27.6% absolute decrease in the 10-year DRFS rate under the same conditions, while the low-risk group remained consistent at a 5.3% ± 12.1% reduction. The effect was more pronounced for high-risk patients with delayed initiation in both discovery and validation sets for both models (**Figures 1**, **2**).

The risk subgroups were also significant in the validation set for Model N°01: Low vs. Intermediate (HR=2.03; 95%CI=1.25-3.31; p=0.005) and Low vs. High (HR=3.50; 95%CI=2.03-6.05; p=<0.001; **Figure 2A**; **Table 4**). However, Model N°02 seems to be a better classifier than the previous one: Low vs. Intermediate (HR=2.48; 95%CI=1.49-4.15; p<0.001) and Low vs. High (HR=4.97; 95%CI=2.79-8.89; p=<0.001; **Figure 2B**; **Table 4**).

## Discussion

We developed a prognostic model using clinicopathological characteristics to estimate the individual patient’s risk of distant recurrence and to identify risk subgroups. The relationship between a delay in TTC and a worse prognosis in TNBC has been shown in previous studies (18, 20, 25). However, heterogeneity among TNBC patients requires individual decisions for treatments. The prognostic model developed in this study is based on clinicopathological characteristics that can be easily found in the clinical records of patients with TNBC, such as age, pT, and pN.

The classification of risk subgroups using prognostic models and the STEPP methodology has been developed for clinical trials and treatments for breast cancer (26, 27). For example, among premenopausal women with HR-positive/HER2-negative early breast cancer, a prognostic model based on clinicopathological characteristics was used to identify high-risk subgroups of recurrence. STEPP analysis demonstrated that high-risk patients experienced an absolute decrease of 5% for 8-year distant recurrence–free interval (DRFI) and 5-year breast cancer–free interval (BCFI) when treated with exemestane+OFS vs. tamoxifen+OFS/ (21, 22). It should be remarked that STEPP is an exploratory tool (28). It means that its application is not limited to clinical trials but rather to determining specific cut-points in the range of values of the covariate of interest (29). Thus, STEPP methodology is an alternative approach to identify interactions between treatment and covariates, define risk subgroups based on a continuous covariate (e.g., risk score/composite risk value), and evaluate the treatment effect (28).

In the discovery set for Model N°01, three significant risk subgroups for distant recurrence were identified: Low (score: 0 – 31), intermediate (score: 32 – 64), and high risk (score: 65 – 100). Regarding Model N°02, three significant risk subgroups were also presented: Low (score: 0 – 26), intermediate (score: 27 – 55), and high risk (score: 56 – 100). Subsequently, the classification of these subgroups was corroborated in the validation set for each model. Our discovery and validation sets showed that the effect in 10-year DRFS of adjuvant chemotherapy initiation >30 days compared to ≤30 days depending on the risk score. According to the TTC, patients classified as low or intermediate risk appeared to have the same effect in 10-year DRFS.In contrast, patients classified as high risk and who start chemotherapy >30 days appear to have a more significant decrease in 10-year DRFS compared to those who began earlier.

In previous studies, no relationship had been found between TTC and worse outcomes when all types of breast cancer were included in the cohort (30, 31). A Kaplan-Meier analysis showed no patterns in delaying the start of chemotherapy for more than four weeks, which indicates no significantly increased hazard ratio in the overall survival (32). Other studies supported that chemotherapy more than 12 weeks from surgery remained significantly associated with inferior survival (33, 34). Meanwhile, another one concluded that a delay of 120 days from diagnosis to chemotherapy was associated with worse outcomes (HR=1.29; 95%CI:1.22–1.37; P<0.001) (35). As has been described, there is no explicit agreement on the ideal time range for TTC so that patients with breast cancer have better outcomes.

However, studies with TNBC patients suggest that 30 days is a meaningful indicator of the quality of cancer care. A recent study reported that adjuvant chemotherapy initiation after 30 days was shown to be associated with a decrease in 10-overall survival in TNBC patients (33). An evaluation of 6827 patients, including TNBC patients and those with HER2+ treated with trastuzumab, showed that patients who started chemotherapy 61 days after surgery had worse survival compared to those who started treatment in the first 30 days after surgery (25). In this context, it appears necessary to make efforts so TNBC patients can start their adjuvant therapies as soon as possible.

Although there has been increased awareness and efforts for health education and promotion in the Latin American region, access to specialized cancer centers and coverage of drug health insurance is limited (36, 37),. This situation hinders the first contact with the health service and the initiation of treatment, which is why the process takes about seven months in countries such as Brazil and Mexico (38). Likewise, a study of Colombian patients associated a higher socioeconomic and educational level with a decreased time to diagnosis and treatment (39).

Regarding adjuvant treatment, the proportion of locally advanced breast cancer (LABC) patients that initiate adjuvant chemotherapy after eight weeks is higher in public hospitals in countries of Latin America such as Bolivia (52.0%), Peru (40.0%), Colombia (25.0%) and Mexico (12.5%), compared to private hospitals in Chile (20.0%) and Peru (20.0%) (40). Furthermore, South America was the region with the most extended approval period for the Adjuvant Treatment Optimization Trial of Lapatinib and Trastuzumab (on average 236 days), while Europe and North America were the fastest (26 days) (41).

The COVID-19 pandemic has highlighted structural problems and inequities in the healthcare system. A multicenter descriptive study showed that, during the pandemic, 51.6% of Peruvian cancer patients experienced a delay in their therapy, 42.5% had their treatment rescheduled, and 30.6% were unable to see a specialist after their diagnosis (42). In Brazil, the trend appears to be similar: 40% of patients reported a delay in cancer treatment or changes in treatment strategy (43–45).

Due to previously mentioned barriers, only 27.5% of Peruvian patients with TNBC had a TTC ≤ 30 days (20). Proper segmentation of patients with TNBC will allow the risk of patients to be evaluated and a treatment strategy that fits their condition. The current study allows physicians to estimate individual risk based on age, pT, and pN to suggest who should urgently start adjuvant chemotherapy.

In conclusion, we developed two predictive models based on age, pT, and pN. Model N°02 was the best one to classify TNBC patients’ candidates for adjuvant chemotherapy into three subgroups: Low (score: 0 – 26), intermediate (score: 27 – 55), and high risk (score: 56 – 100). High-risk patients with TNBC who started their treatment >30 days experienced a decrease from 75.0% to 21.6% in 10-year DRFS, compared to those who started<30 days. This trend was confirmed in the validation cohort (58.8% to 36.1%). Thus, we suggest using our model to bring additional efforts to high-risk patients with TNBC to avoid increasing their relapse probabilities.

## Data availability statement

The original contributions presented in the study are included in the article/**Supplementary Material**. Further inquiries can be directed to the corresponding author.

## Ethics statement

The study was approved by the Ethics Review Board of the Instituto Nacional de Enfermedades Neoplasicas (INEN 22-03) and conducted in compliance with all relevant ethical guidelines.

## Author contributions

Conception and design: HG, YF, JP, ZM. Administrative support: ZM, YF. Collection and assembly of data: ZM. Data analysis and interpretation: ZM, YF, JP, HG. Manuscript writing: All authors. Final approval of manuscript: All authors. Accountable for all aspects of the work: All authors. All authors contributed to the article and approved the submitted version.

## Publisher’s notes

All claims expressed in this article are solely those of the authors and do not necessarily represent those of their affiliated organizations, or those of the publisher, the editors and the reviewers. Any product that may be evaluated in this article, or claim that may be made by its manufacturer, is not guaranteed or endorsed by the publisher.
