# Supplementary material for: Subpopulation treatment effect pattern plot analysis: a prognostic model for distant recurrence-free survival to estimate delayed adjuvant chemotherapy initiation effect in triple-negative breast cancer
Source: Front Oncol. 2023 Nov 6;13:1193927. doi: 10.3389/fonc.2023.1193927 (PMC10657890; doi:10.3389/fonc.2023.1193927)

Fig. S1: Log-Log Kaplan-Meier estimates of Distant Recurrence-Free Survival (DRFS) interval in discovery cohort according to three clinicopathologic characteristics. (A) Age group 1, (B) Age group 2, (C) pT, (D) pN

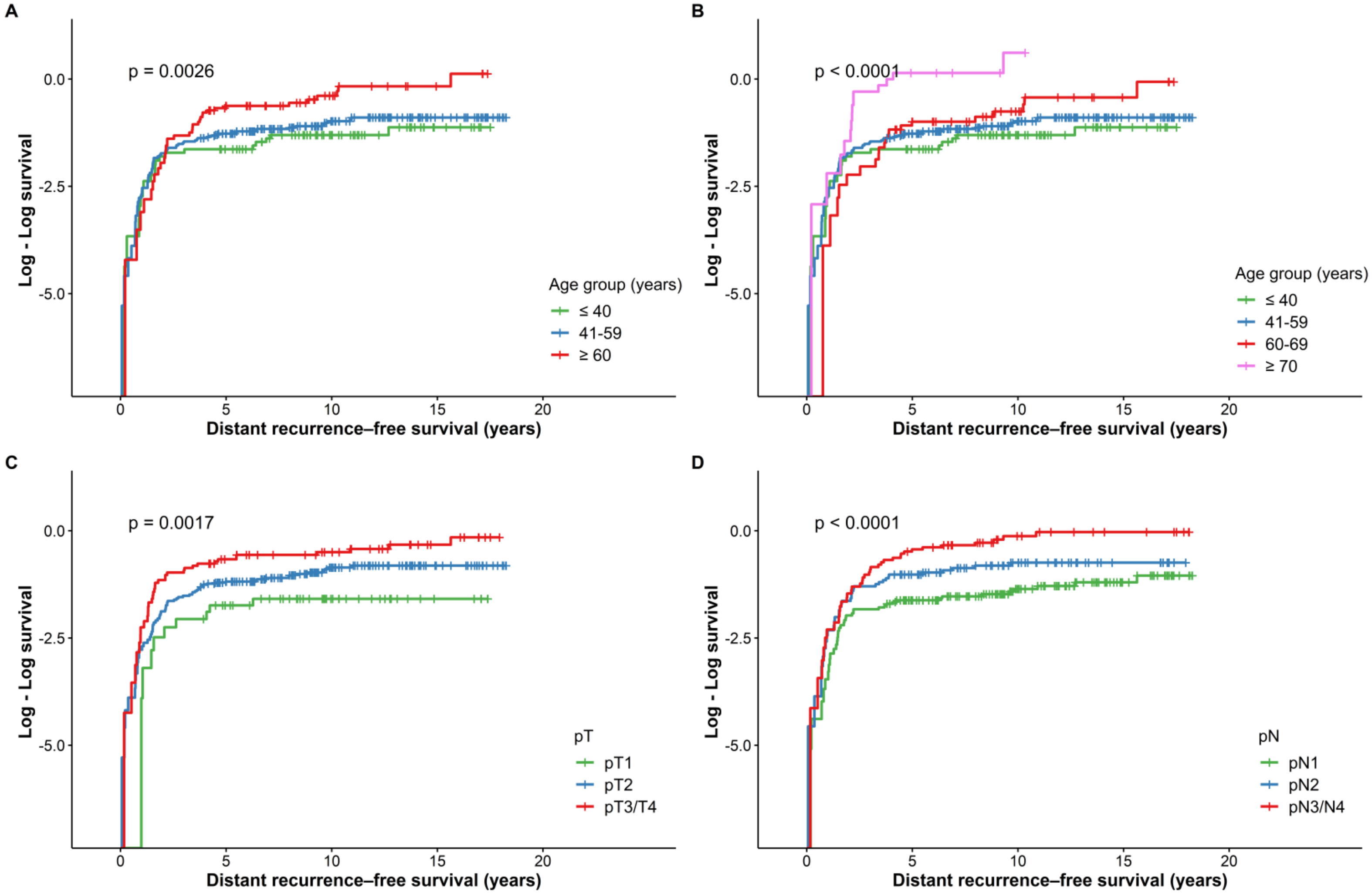

Supplement: Supplementary file 1 [file Image_1.pdf]
